# Development of an oligonucleotide-based fluorescence assay for the identification of tyrosyl-DNA phosphodiesterase 1 (TDP1) inhibitors

**DOI:** 10.1016/j.ab.2014.03.004

**Published:** 2014-06-01

**Authors:** Sarah Walker, Cornelia Meisenberg, Rachel A. Bibby, Trevor Askwith, Gareth Williams, Frauke H. Rininsland, Laurence H. Pearl, Antony W. Oliver, Sherif El-Khamisy, Simon Ward, John R. Atack

**Affiliations:** a Translational Drug Discovery Group, School of Life Sciences, University of Sussex, Brighton BN1 9QJ, UK; b Wellcome Trust DNA Repair Group, Genome Damage and Stability Centre, University of Sussex, Brighton BN1 9RQ, UK; c Cancer Research UK DNA Repair Enzymes Group, Genome Damage and Stability Centre, University of Sussex, Science Park Road, Brighton BN1 9RQ, UK; d Gyrasol Technologies, 2029 Becker Drive, Lawrence, KS 66047, USA; e Mammalian Genome Stability Group, Kreb’s Institute, University of Sheffield, Firth Court, Western Bank, Sheffield S10 2TN, UK

**Keywords:** TDP1, Assay, High-throughput screen, Fragment, Inhibitor, Topoisomerase

## Abstract

Topoisomerase 1 (TOP1) generates transient nicks in the DNA to relieve torsional stress encountered during the cellular processes of transcription, replication, and recombination. At the site of the nick there is a covalent linkage of TOP1 with DNA via a tyrosine residue. This reversible TOP1-cleavage complex intermediate can become trapped on DNA by TOP1 poisons such as camptothecin, or by collision with replication or transcription machinery, thereby causing protein-linked DNA single- or double-strand breaks and resulting in cell death. Tyrosyl-DNA phosphodiesterase 1 (TDP1) is a key enzyme involved in the repair of TOP1-associated DNA breaks via hydrolysis of 3′-phosphotyrosine bonds. Inhibition of TDP1 is therefore an attractive strategy for targeting cancer cells in conjunction with TOP1 poisons. Existing methods for monitoring the phosphodiesterase activity of TDP1 are generally gel based or of high cost. Here we report a novel, oligonucleotide-based fluorescence assay that is robust, sensitive, and suitable for high-throughput screening of both fragment and small compound libraries for the detection of TDP1 inhibitors. We further validated the assay using whole cell extracts, extending its potential application to determine of TDP1 activity in clinical samples from patients undergoing chemotherapy.

DNA Topoisomerase 1 (TOP1)^1^ generates a transient nick in the DNA in order to enable relaxation of torsional stress, exerted on the DNA during transcription and replication [1]. During this process, the hydroxyl group of TOP1 Tyr723 becomes covalently bound to the 3′-phosphate group of a DNA nick [2]. This TOP1-DNA intermediate is normally transient and religation occurs rapidly on resolution of supercoiling. However, when TOP1 encounters endogenous DNA lesions such as abasic sites or base damage, or when cells are exposed to drugs such as the TOP1 poison camptothecin (CPT), TOP1 can stall on the DNA [2]. These stalled TOP1-DNA intermediates are termed cleavage complexes (TOP1-CC). TOP1-CC may block the replicative or transcriptional polymerases, and result in the formation of single-strand breaks (SSB) or double-strand breaks (DSB) with TOP1 linked covalently to the 3′-ends [3]. Cell death can result if these lesions are not efficiently repaired.

Repair of TOP1-CC is initiated by the degradation of the covalently linked TOP1 by ubiquitin-mediated proteolysis [4]. Tyrosyl-DNA phosphodiesterase 1 (TDP1) subsequently catalyses the hydrolysis of 3′-phosphodiester bonds between a tyrosine residue and the 3′-DNA phosphate [5]. TDP1 belongs to the phospholipase D superfamily of enzymes that contain two conserved HKD motifs [6]. These motifs contain conserved residues that cluster together within the active site. Mutation of either histidine 263 or 493 within the conserved HKD motif results in loss of activity, demonstrating that these residues are critical for TDP1 activity [6].

It is worth highlighting that TDP1 is a broad-spectrum 3′-DNA end-processing enzyme. For example, it can process 3′-phosphoglycolate ends on DSBs produced by ionising radiation or radiomimetic drugs [7] and can cleave apurinic/apyrimidinic site (AP) sites induced by alkylating agents used in cancer therapy such as temozolomide [8,9]. Point mutations in the TDP1 catalytic site (H493R) cause SCAN1 neurological disorder (spinocerebellar ataxia with axonal neuropathy) where patients present with cerebellar atrophy and peripheral neuropathy [10]. Cell lines derived from SCAN1 patients accumulate Top1-SSBs due to defects in SSB repair and they are hypersensitive to CPT [11,12]. TDP1 is subject to posttranslational modifications that control its cellular activity such as phosphorylation [13,14] and SUMOylation [15]; however the contribution of these modifications to TDP1 cellular function in cancer remains elusive.

Overexpression of TDP1 increases resistance to CPT [16,17] and increased expression of TDP1 protein resulting in increased enzyme activity has been observed in a set of non-small-cell lung cancer samples [18]. Increased expression of TDP1 has also been observed in a panel of colorectal tumor specimens [2]. TDP1 inhibitors could therefore have broad clinical utility both as a single-agent treatment in tumors with genetic backgrounds that are sensitive to TDP1 inhibition and as a combination therapy in conjunction with established TOP1 poisons, radiotherapy, or temozolomide.

Current assays for the assessment of the tyrosyl DNA phosphodiesterase activity of TDP1 *in vitro* have utilised a chromogenic substrate [19], a biotinylated system [20], or an Alphascreen format [21]. Elegant studies have recently reported the use of oligonucleotide substrates to monitor the AP-site cleavage activity of TDP1 [22] and as real-time biosensors in cellular material [23]. Here we focused on determining the tyrosyl DNA phosphodiesterase activity of TDP1, which is the preferred activity *in vivo* and *in vitro*, and is likely to be exploited in conjunction with camptothecin-derived protocols for cancer therapy. A radiolabeled gel assay has been widely adopted in the past but can be costly and time-consuming, and is therefore not applicable to high-throughput screening regimens. Previously reported chromogenic and biotinylated assays utilised physiologically less relevant substrates and high enzyme concentrations. Here, we describe the development of a robust, sensitive, more cost efficient assay for high-throughput screening of fragment and small compound libraries to identify novel TDP1 inhibitors.

## Materials and methods

### Cloning and purification of recombinant human TDP1

A codon-optimised synthetic gene, encoding the catalytic domain of human TDP1, was purchased from GenScript USA Inc. Details of the expression construct are consistent with that described in Interthal et al. [6]. Recombinant protein was expressed in, then purified from *Escherichia coli* strain Rosetta2 (DE3) (Merck, Darmstadt, Germany) using standard chromatography techniques, following the protocol described by Interthal et al. [6].

### Preparation of oligonucleotide substrates

Oligonucleotides of 13mer with a 3′-phosphotyrosyl bond conjugated with an FITC molecule were purchased from Midland Certified Reagent (Midland, TX, USA).

### Development of the TDP1 fluorescence assay

The assay was developed in 384-well black plates in a final reaction volume of 15 μL. The final concentration of assay buffer was 50 mM Tris 8.0, 5 mM MgCl_2_, 80 mM KCl, 0.05% Tween 20, and 1 mM DTT. Reactions were initiated by addition of substrate and plates were incubated at 25 °C. Final concentrations of 6.25 pM TDP1 and 10 nM substrate were used for routine screening. Addition of quench reagent (2 μL sensor + 30 μL enhancer buffer) (Gyrasol Technologies, KS, USA) stopped the reaction and fluorescence was subsequently monitored with excitation and emission wavelengths of λ_ex_ 490 nm and λ_em_ 520 nm using a BMG Labtech Pherastar plate reader. *In vitro* 3′-tyrosyl-DNA phosphodiesterase 1 activity of whole cell extracts (WCE) was determined using the TDP1 fluorescence assay format in a 15 μL reaction volume. Final concentrations of WCE (ng/μg) per well, diluted in assay buffer, were incubated with 10 nM final concentration of oligonucleotide substrate.

### Data analysis

Data were analysed using GraphPad Prism (GraphPad Software Inc., La Jolla, CA). The affinity of the substrate for the enzyme, the Michaelis constant (or *K*_m_ value), was determined according to the Michaelis–Menten equation: *Y* = *V*_max_ * *X*/(*K*_m_ + *X*). The catalytic (or first-order rate) constant *K*_cat_ was calculated using the equation: (*Y* = *E*_t_ * *k*_cat_ *X/(*K*_m_ + *X*)). IC_50_ values were generated by transforming the data (*X* = Log(*X*)) and then fitting these data to the equation: *Y* = Bottom + (Top–Bottom)/(1 + 10^((LogEC50^ ^−^ *^X^*^)^ ^∗^ ^HillSlope)^).

As an index of assay reproducibility, the statistical parameter *Z*-prime (*Z*′) was calculated according to Zhang et al. [24].

### Fragment library single point screening

A library of 1500 fragments (Maybridge, Thermo Fisher Scientific, UK) was screened, at a single point concentration of 1 mM using the TDP1 fluorescence assay as described.

### Cell culture

HEK293 cells were maintained at 5% CO_2_, in DMEM (Life Technologies, Paisley, UK) supplemented with 10% FCS, 100 U penicillin, 100 μg streptomycin, and 2 mM l-glutamine. TDP1^−/−^ mouse embryonic fibroblasts (MEF) [14,25,26] were maintained at 2% O_2_ in DMEM (Life Technologies, Paisley, UK) supplemented with 15% FCS, 2 mM l-glutamine, 100 U penicillin, 100 μM nonessential amino acids, and 100 μg streptomycin. TDP2^−/−^ DT40 cells containing either a pcDNA3.1-HisC empty vector or pcDNA3.1-HisC encoding human TDP2 were [27,28] maintained at 39 °C, 5% CO_2_, in RPMI (Life Technologies, Paisley, UK) supplemented with 10% FCS, 1% chicken serum, 100 U penicillin, 100 μg streptomycin, 2 mM l-glutamine, and 50 μM β-mercaptoethanol. For transient transfection experiments, HEK293 cells were transfected with pCI empty vector or pCI encoding human TDP1 [15] using Lipofectamine LTX with Plus transfection reagent (Life Technologies, Paisley, UK), according to the manufacturer’s instructions. Cell pellets were harvested 24 h post transfection and stored at −80 °C.

### Whole cell extract preparation

Cell pellets were resuspended in ice-cold lysis buffer (20 mM Tris–HCl, pH 7.5, 10 mM EDTA, pH 8.0, 100 mM NaCl, 1% Triton X-100) containing 1× final concentration of complete mini EDTA-free protease inhibitor cocktail (Roche Applied Science, Burgess Hill, UK) at a 1:4 pellet volume to lysis buffer ratio. Pellets were extracted for 30 min at 4 °C and the suspension was centrifuged at 13,000 rpm for 10 min at 4 °C. Supernatant was collected and the protein concentration determined by Bradford assay prior to aliquoting and storing at −80 °C.

### Western blotting

Whole cell extract (40 μg) was separated by 10% SDS-polyacrylamide gel electrophoresis (PAGE) and transferred on to a Hybond-C Extra nitrocellulose membrane (Fisher Scientific UK, Loughborough, UK). The membrane was blocked in 5% PBS-milk for 1 h prior to immunoblotting. Antibodies against TDP1 (ab4166; Abcam, Cambridge, UK) and TDP2 [22] were used overnight at a 1:2000 dilution in 5% PBST-milk. Actin antibodies (A4700; Sigma, Gillingham, UK) were used at 1:3000 dilution in 5% PBST-milk for 1 h. HRP-labeled polyclonal rabbit anti-mouse and polyclonal goat anti-rabbit secondary antibodies were used at a 1:3000 dilution in 5% PBST-milk and were obtained from Dako (Ely, UK). Blots were developed using the chemiluminescent detection reagent, SuperSignal West Pico chemiluminescent substrate (Fisher Scientific UK, Loughborough, UK).

## Results and discussion

**Fig. 1:**
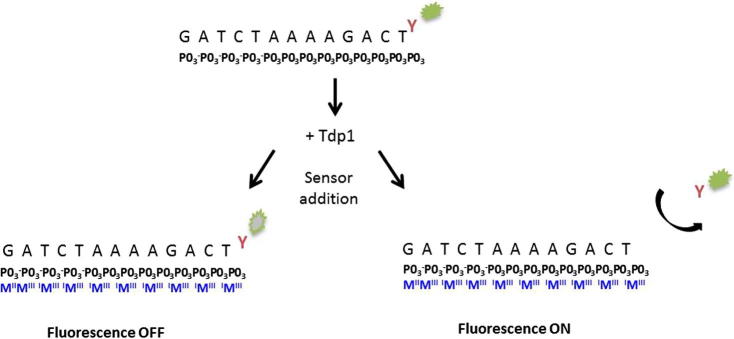
Gyrasol assay technology. A 13mer oligo with a 3′-tyrosine-conjugated FITC molecule was produced. The phosphodiester bond between the tyrosine and the DNA can be hydrolysed by tyrosyl-DNA-phosphodiesterase 1 (TDP1). Addition of a small molecular, nonfluorescent trivalent metal ion sensor (MIII) (Gyrasol Technologies, USA) binds to the phosphate bone of the ss-DNA oligo. The fluorescence of any fluor in close proximity to the sensor is quenched by electron transfer, while any fluor separated from the DNA (∼1 nm; 10 A) is too distant for electron transfer quench.

Here we describe the combination of Gyrasol assay technology (Fig. 1) with a fluorescent oligonucleotide substrate in order to identify novel TDP1 inhibitors. Gyrasol assay technology employs the use of a small molecular, nonfluorescent trivalent metal ion sensor to bind to the phosphate backbone of the DNA oligonucleotide. The fluorescence of any fluorophore in close proximity to the sensor is quenched by electron transfer. Any fluorophore that is separated from the DNA by a distance of greater than approximately 1 nm is too distant for electron transfer quench. The action of TDP1 on the phosphotyrosine moiety on the oligonucleotide substrate cleaves the FITC molecule. The increased distance of cleaved FITC molecules from the DNA prevents quenching and therefore specific tyrosyl DNA phosphodiesterase activity of TDP1 can be monitored as an increase in fluorescence intensity.

**Fig. 2:**
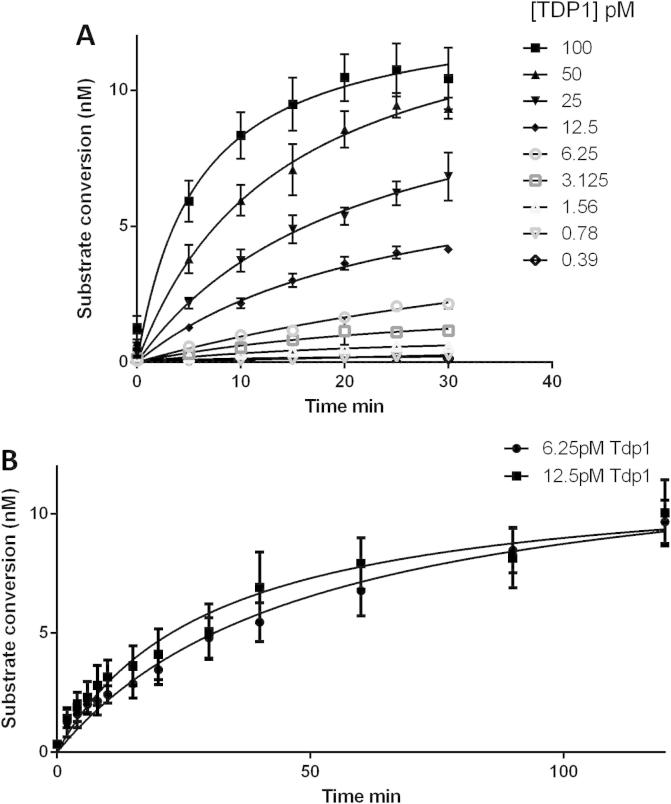
Enzyme kinetics evaluation. (A) Enzyme titration and time course of TDP1 in the Gyrasol assay. Final substrate concentration was 10 nM. (B) Kinetics of product formation for two TDP1 concentrations. Final substrate concentration was 10 nM. Graphs represent the mean of three independent experiments. Error bars represent the standard deviation.

The ability of WT TDP1 to cleave the phosphotyrosine bond present on the fluorescent-tagged oligonucleotide was determined at different enzyme concentrations over a short time course (Fig. 2A). Two concentrations from this initial time course were then examined in detail in a 3-time course experiment (Fig. 2B). These data demonstrated that the assay signal was dependent on both time and enzyme concentration. Readings before addition of quench reagent showed that fluorescence readings in the absence of quench reagent were uniform across the plate (∼4000 RFU for 10 nM substrate). On addition of quench reagent consistent background and activity fluorescence readings were obtained for wells with or without TDP1, indicating that the fluorescence readings obtained were specific for TDP1 activity (data not shown). The enzyme concentration employed for further optimisation of the assay was 6.25 pM, reflecting the efficient turnover of the oligonucleotide substrate by TDP1.

**Fig. 3:**
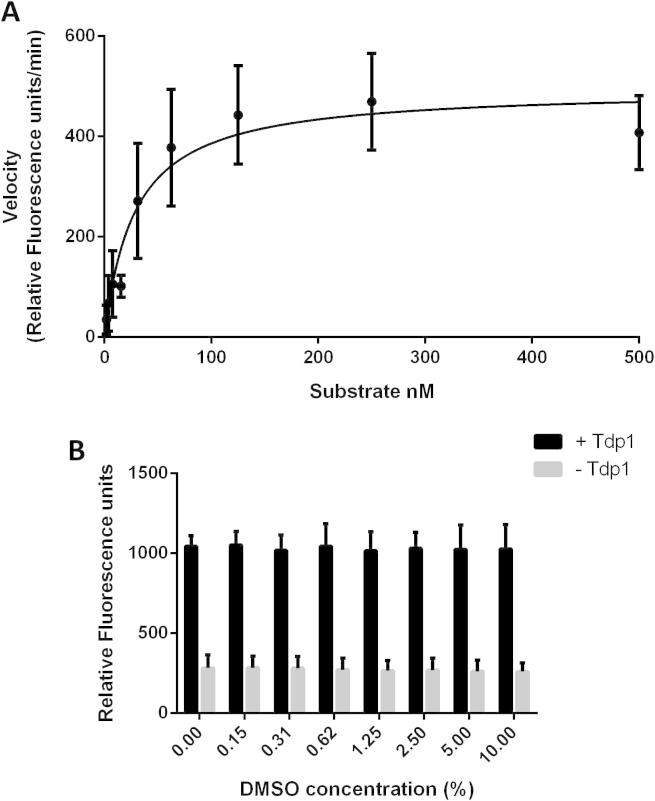
*K*_m_ determination and DMSO tolerance of TDP1. (A) 6.25 pM TDP1 was incubated with increasing substrate concentrations for 10 min prior to addition of quench reagent. Fluorescence signal was measured and the rate of product formation calculated. Data were fitted to the Michaelis–Menten equation using GraphPad Prism. Graph represents the mean of three independent experiments. Error bars represent the standard deviation. (B) A 6.25 pM TDP1 was incubated with increasing concentrations of DMSO for 15 min prior to incubation with 10 nM oligonucleotide substrate for 10 min. Quench reagent was then added and fluorescence signal measured. Graph represents the mean of three independent experiments. Error bars represent the standard deviation.

The *K*_m_ for the oligonucleotide substrate was calculated to be 49 ± 8 nM (Fig. 3A) under the assay conditions employed and the *K*_cat_ was determined to be 107.1 ± 5.4 min^−1^. These data are in agreement with published studies of TDP1 activity on single-stranded oligonucleotide substrates [29]. A final concentration of 10 nM oligonucleotide substrate was selected for use in fragment library screening. This concentration provided a good signal to noise ratio while using minimal substrate to enable the assay to remain cost-effective to run.

Using the conditions described above, the assay was linear for approximately 30 min. For screening purposes, the reaction was incubated for 10 min prior to the addition of quench reagent and immediate fluorescence monitoring. Under these conditions a signal to noise ratio of 3:1 was achieved. While the addition of quench appeared to stop the reaction, further incubation of the plate in the presence of the quench reagent caused increased background readings, potentially due to precipitation of quenching reagents.

In order to screen potential inhibitors of TDP1, the tolerance of the enzyme to DMSO was determined. Under these assay conditions, the enzyme was found to be tolerant to high levels of DMSO (10%) with little or no loss of signal (Fig. 3B).

**Fig. 4:**
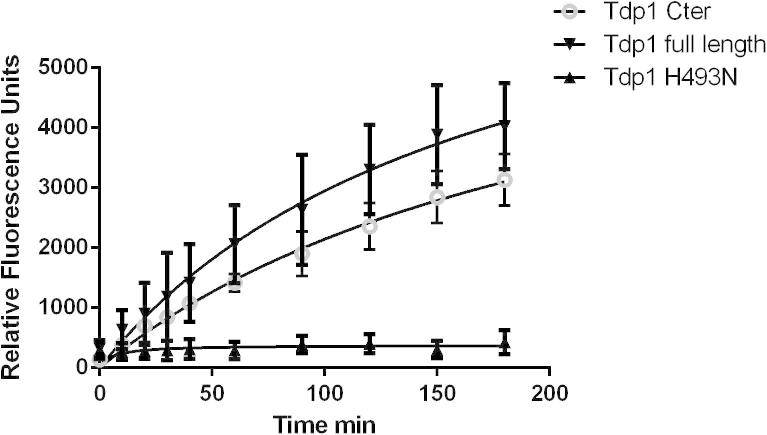
TDP1 mutant proteins. Recombinant mutant TDP1 proteins (6.25 pM) as described in the text were incubated with a final concentration of 10 nM oligonucleotide substrate over a 3-h time course. Quench reagent was then added and fluorescence signal measured. Graph represents the mean of three independent experiments. Error bars represent the standard deviation.

To further validate the assay a range of recombinant mutant TDP1 proteins were tested at the same concentration under the optimised assay conditions described. Recombinant proteins tested included full-length TDP1, C terminal TDP1 containing the catalytic domain only, and mutants of the S81 phosphorylation site [14] and the K111 SUMOylation site [15]. All recombinant mutant proteins were found to be active in the assay (data not shown), with the exception of the H493N mutant protein. This mutation recapitulates the H493R mutation identified in SCAN1 patients where the enzyme loses more than 95% of its catalytic activity [30]. As expected, little to no turnover of the substrate was observed on incubation of the substrate with TDP1 H493N (Fig. 4).

**Fig. 5:**
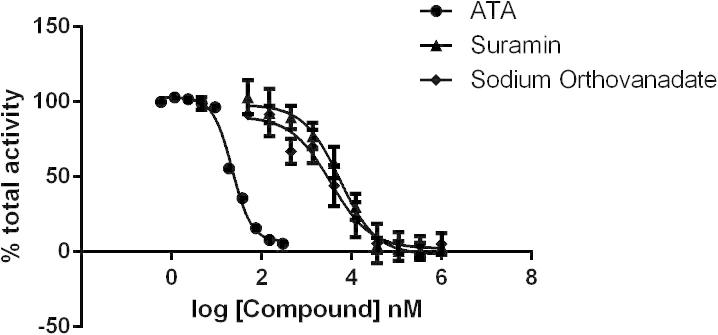
Testing of reference inhibitors. Inhibition of TDP1 by suramin, sodium orthovanadate, and ATA as determined in the Gyrasol assay.

A number of reference TDP1 inhibitory compounds were tested to verify that the assay is suitable for screening novel inhibitors. Suramin, sodium orthovanadate, and aurintricarboxylic acid (ATA) were all found to inhibit Tdp1 in this assay with IC_50_ values of 5 μM, 4 μM, and 22 nM, respectively (Fig. 5). The IC_50_ data obtained for suramin and ATA using this assay are in agreement with published data [22].

Importantly, the enzyme and substrate concentrations described above are significantly lower than those of previously described formats [20,21], demonstrating the improved sensitivity of this assay. The low enzyme and substrate concentrations employed enable an extremely cost effective approach to the screening of large compound libraries.

A library of 1500 fragments was then screened at a single concentration of 1 mM. Fragments are expected to be weak enzyme inhibitors and are therefore commonly screened at a higher concentration than standard compound libraries [31,32]. The plate *Z* factor is a statistical measurement calculated from the means and variance of both the negative and the positive controls on each plate. A plate *Z* factor of above 0.5 indicates an excellent assay for screening purposes [24]. Plate *Z* factors calculated from the negative (−TDP1) and positive (+TDP1) control wells on each screening plate were routinely observed to be >0.5 (data not shown), although any plate with a *Z* factor of >0.4 was accepted. The TDP1 fluorescence assay was found to be extremely sensitive, robust and highly suitable for screening of inhibitors for TDP1 3′-phosphodiesterase activity.

The determination of TDP1 activity in patient cell preparations could have important clinical applications in cancer patients. Therefore we tested the ability of the TDP1 fluorescence assay to determine TDP1 activity levels in whole cell extracts from cells.

**Fig. 6:**
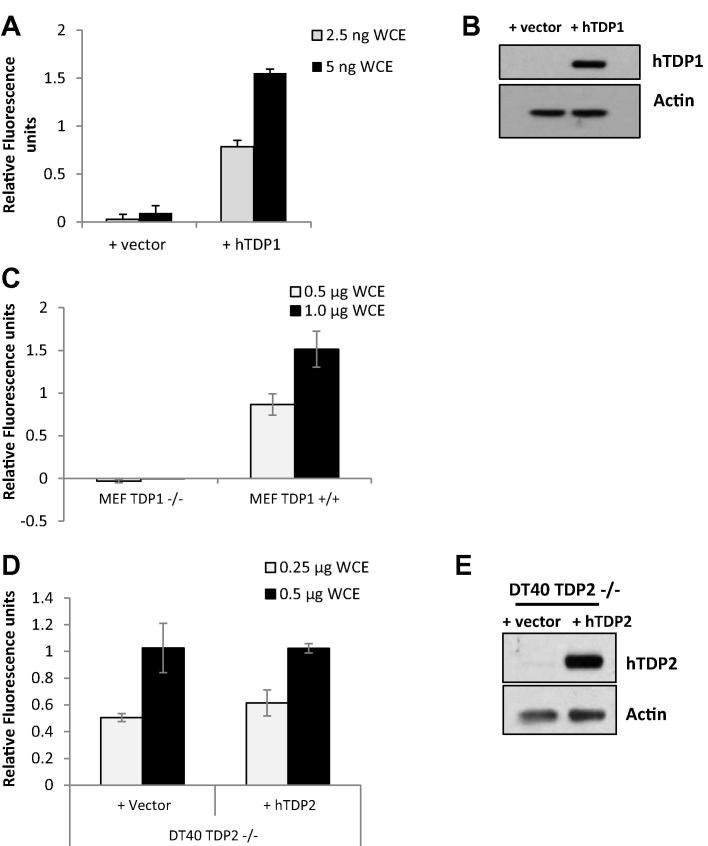
TDP1 activity in whole cell extracts. Whole cell extracts (WCE) generated from HEK293 transfected with pCI empty vector or pCI vector for human TDP1 overexpression (A); wild-type or TDP1^−/−^ mouse embryonic fibroblast (MEF)(C); and TDP2^−/−^ DT40 cells containing either pcDNA3.1-HisC empty vector or pcDNA3.1-HisC vector for human TDP2 overexpression (D) were used as the TDP1 source in the TDP1 fluorescent assay. The 15 μL reactions contained the indicated amounts of WCE. The reaction was carried out at room temperature for 10 min and terminated by addition of quench reagent. Relative fluorescence is shown and was calculated against the fluorescence obtained for 6.25 pM recombinant WT human TDP1 protein for each of three independent experiments. Error bars represent the standard deviation. WCEs generated from HEK293 transfected with pCI empty vector or pCI vector for human TDP1 overexpression (B) and TDP2^−/−^ DT40 containing either pcDNA3.1-HisC empty vector or pcDNA3.1-HisC vector for human TDP2 expression (E) were separated by 10% SDS-PAGE, followed by Western blotting analysis using antibodies against human TDP2 and actin.

Whole cell extracts were generated from HEK293T cells transfected with pCI vector for stable overexpression of human TDP1, wild-type, and TDP1^−/−^ mouse embryonic fibroblasts (MEF) and from TDP2^−/−^ DT40 transfected with pcDNA3.1-HisC vector for human TDP2 overexpression [27]. WCE were then used as the TDP1 source in the TDP1 fluorescence assay. Increased TDP1 activity was observed in HEK293T WCE overexpressing TDP1 compared to HEK293T WCE containing empty vector (Fig. 6A and B). Increased TDP1 activity was observed in the TDP1^+/+^ MEF WCE compared to negligible activity in the TDP1^−/−^ MEF WCE (Fig. 6C). Similar levels of activity were observed in TDP2^+/+^ and ^−/−^ DT40 cells, demonstrating that TDP2 is not able to contribute to cleavage of the 3′-phosphotyosine substrate under these conditions (Fig. 6D and E). These results indicate that the activity observed is related to the TDP1 cellular expression levels and that this assay may be used to assess TDP1 activity from mouse, human, and chicken material. The WCE assay could therefore be a useful tool for confirming that inhibitors of TDP1 identified in biochemical screens can also inhibit cellular TDP1. The specificity that we have observed also indicates that the WCE assay could potentially be used as a diagnostic tool for determination of TDP1 activity directly in cancer cells or tissues from patients, thus identifying those patients that could benefit from treatment with TDP1 inhibitors. However, further work would need to be undertaken to confirm this application.

The identification of TDP1 inhibitors could have important clinical applications both in those patients where resistance to existing treatments such as CPT is challenging and also potentially as enhancers to radiotherapy treatments. Utilisation of biochemical assays combined with a fragment-based drug design method to synthesize new molecules could be a powerful approach to the discovery of novel oncology therapies.

## Footnotes

1 *Abbreviations used:* ATA, aurintricarboxylic acid; CPT, camptothecin; DSB, double-strand breaks; SSB, single-strand breaks; TDP1, tyrosyl-DNA-phosphodiesterase 1; TOP1, Topoisomerase 1; TOP1-CC, TOP1-DNA intermediates are termed cleavage complexes; WCE, whole cell extracts.
